# Is Penicillin-Nonsusceptible *Streptococcus pneumoniae* a Significant Challenge to Healthcare System? A Systematic Review and Meta-Analysis

**DOI:** 10.1155/2021/5573345

**Published:** 2021-05-27

**Authors:** Farzad Khademi, Amirhossein Sahebkar

**Affiliations:** ^1^Department of Microbiology, School of Medicine, Ardabil University of Medical Sciences, Ardabil, Iran; ^2^Applied Biomedical Research Center, Mashhad University of Medical Sciences, Mashhad, Iran; ^3^Biotechnology Research Center, Pharmaceutical Technology Institute, Mashhad University of Medical Sciences, Mashhad, Iran; ^4^School of Pharmacy, Mashhad University of Medical Sciences, Mashhad, Iran

## Abstract

**Background:**

In recent years, antibiotic-resistant pathogens including penicillin-nonsusceptible *Streptococcus pneumoniae* (PNSP) have posed serious threats against human health. The aim of this meta-analysis was to investigate the prevalence of *Streptococcus pneumoniae* drug resistance particularly the incidence of PNSP strains in Iran.

**Methods:**

A systematic search was done in national and international electronic databases using Persian and English keywords. Up until May 20, 2020, a total of 58 publications were detected as eligible articles based on the inclusion and exclusion criteria, and then the selected studies were enrolled for data extraction and meta-analysis according to the PRISMA guidelines.

**Results:**

A high rate of PNSP (46.9%) and multidrug-resistant (MDR) *S. pneumoniae* (45.3%) in our isolates were evident. Furthermore, total frequency resistance to other drugs in *S. pneumoniae* was as follows: erythromycin 41.1%, azithromycin 53.2%, tetracycline 39.9%, levofloxacin 1.7%, rifampin 1.2%, clindamycin 31.7%, vancomycin 1.7%, trimethoprim/sulfamethoxazole 63.9%, chloramphenicol 20%, ceftriaxone 10.9%, amoxicillin 30.5%, ciprofloxacin 8.3%, imipenem 6.1%, linezolid 0%, and cefotaxime 8.3%.

**Conclusion:**

Although the overall prevalence of cephalosporin- and carbapenem-resistant *Streptococcus pneumoniae* was low, penicillin-resistant strains, especially PNSP, could become a significant challenge to the healthcare system in Iran. Hence, the prescription of penicillin as the first-choice antibiotic in the treatment of *S. pneumoniae* infections should be avoided.

## 1. Introduction

*Streptococcus pneumoniae* (*S. pneumoniae*) (pneumococcus) is a Gram-positive diplococcus that is an exclusively normal inhabitant in the oropharynx and nasopharynx of healthy individuals [1, 2]. Colonization rates are higher in the extreme of age (children under 5 and adults older than 65 years old) and immunocompromised patients especially in developing countries [3]. The bacterium enters the body through droplets and aerosols by person-to-person transmission. It then disseminates into other sites including circulation, brain, lungs, paranasal sinuses, and middle ears and causes severe diseases such as pneumonia, sinusitis, otitis media, bacteremia, and meningitis [1–3]. Pneumonia of any cause is an important disease affecting children under the age of five. In 2017, the World Health Organization (WHO) announced 808,694 pneumonia-related child deaths which accounts for 15% of total mortality in children younger than five years old [4]. The two most common bacterial causes of pneumonia in children are *S. pneumoniae* and *Haemophilus influenzae* type b, respectively [3]. Recently, a plan released by the WHO/UNICEF named the Integrated Global Action Plan for the Prevention and Control of Pneumonia and Diarrhea (GAPPD) with the aim of reducing the death rate to less than 3 children per 1000 live births by 2025 [4]. The main strategies to protect, prevent, and treat children with pneumococcal pneumonia include exclusive breastfeeding, adequate complementary feeding, hand washing, reducing household air pollution, prevention of HIV, oxygen therapy, vaccinations, and also the administration of appropriate antibiotics [4]. Potent anticapsular pneumococcal vaccines (PPSV23, PCV13, and PCV7) were developed based on the prevalent serotypes of *S. pneumoniae*; however, they are less effective in developing countries due to different distribution patterns of serotypes by geographic locations [1], and hence, they are not part of the childhood immunization plan in Iran. Over the last several decades, penicillin was the drug of choice for the treatment of pneumococcal diseases [1, 2]. However, up to 40% of these bacteria are found to be penicillin-resistant [5]. A list of antibiotic-resistant pathogens was released by WHO in February 2017 that urgently requires effective antibiotics [6]. Penicillin-nonsusceptible *S. pneumoniae* (PNSP) strains are priority 3 (medium) on the list and known as increasingly drug-resistant pathogens which require further research and development of new antibiotics [6]. Given the distinct geographical distributions, which can affect bacterial phenotypic and genetic characteristics such as drug susceptibility, and self-medication of antibiotics in Iran, the current systematic review and meta-analysis was performed to follow four objectives: (1) to estimate the overall prevalence of *S. pneumoniae* strains resistant to different antibiotics among all age groups in Iran, (2) to determine *S. pneumoniae* drug resistance in Iranian children, (3) to assess the prevalence of PNSP strains, and (4) to investigate antimicrobial resistance profiles in different provinces of Iran.

## 2. Methods

### 2.1. Search Strategies

The current systematic review and meta-analysis is designed based on the PRISMA (Preferred Reporting Items for Systematic Reviews and Meta-Analysis) guidelines [7]. A comprehensive search was performed on studies published from October 1993 to May 2020. English keywords in the ISI Web of Knowledge, PubMed, Scopus, and Google Scholar databases and Persian keywords in national search engines including Scientific Information Database (http://www.sid.ir) and Magiran (http://www.Magiran.com) were used to find original articles addressing *S. pneumoniae* antibiotic resistance in Iran. For this purpose, the search terms (i.e., *S. pneumoniae*, antibiotic resistance, and Iran) were extracted from Medical Subject Headings (MeSH) and combined with connectors (AND/OR).

### 2.2. Inclusion and Exclusion Criteria

The articles were selected based on the title, abstract, and full text. First, the titles of cross-sectional studies on the prevalence of drug resistance were evaluated according to the author, bacterium, and country names, and then, abstracts and full texts were further assessed. Inclusion criteria were original articles assessing the prevalence of pneumococcus drug resistance, full-text availability, publication in English or Persian languages, and studies with sufficient data and limited to Iran. Exclusion criteria were studies reporting drug resistance patterns only at the level of *Streptococcus* genus or other than *S. pneumoniae*, evaluating the prevalence of *S. pneumoniae* resistance with low sample size, repetitive publications, nonoriginal articles, and articles available only in abstract form or abstracts from conferences.

### 2.3. Quality Assessment and Data Extraction

Included articles were further assessed in terms of quality using the Joanna Briggs Institute (JBI) critical appraisal checklist, and then, necessary data were extracted and tabulated in Table 1 [66]. The main data included the first author's surname, study location, publication date, study enrollment date, age group, sample size, antibiotic susceptibility testing methods, the prevalence of *S. pneumoniae* resistance to different drugs, and the prevalence of multidrug-resistant (MDR) pneumococci.

### 2.4. Meta-Analysis

Meta-analysis of the extracted data on the *S. pneumoniae* antibiotic resistance was performed using the Comprehensive Meta-Analysis (CMA) software (Biostat, Englewood, NJ), and the frequency of drug resistance was expressed as the percentage and 95% confidence intervals (95% CIs). Further analysis on the location of the study and age groups was also conducted. To evaluate the heterogeneity across the included studies, *I*^2^ statistics and the chi-square test (*χ*^2^) with the Cochrane *Q* statistic (*Q* test) (*p* value <0.05 was considered statistically significant) were used. A random-effects model (DerSimonian–Laird method) was used to pool the data when heterogeneity was considered high (*I*^*2*^ ≥ 25%). Distribution bias among published studies was calculated quantitatively using Begg's and Egger's tests (*p* value <0.05 indicates a significant bias) and visualized via the funnel plot graphs for each antibiotic.

## 3. Results

### 3.1. Results of Search and Characteristics of the Included Articles

**Figure 1:**
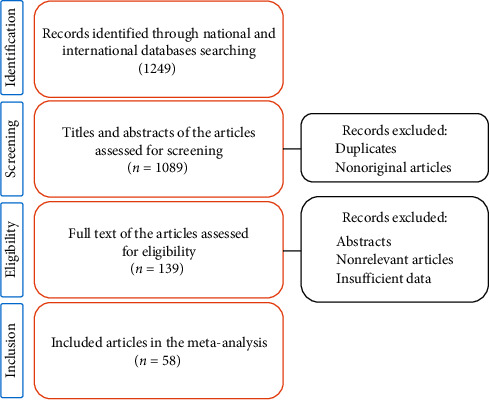
Flow diagram of literature search and study selection.

Data were available from 15 provinces as follows: Ardabil (*n* = 1), Chaharmahal and Bakhtiari (*n* = 2), East Azerbaijan (*n* = 1), Fars (*n* = 6), Golestan (*n* = 1), Hamadan (*n* = 5), Isfahan (*n* = 2), Kermanshah (*n* = 1), Khuzestan (*n* = 1), Qazvin (*n* = 1), Tehran (*n* = 31), Sistan and Balouchastan (*n* = 3), West Azerbaijan (*n* = 1), Yazd (*n* = 1), and Zanjan (*n* = 1). Detailed characteristics of the selected articles are summarized in Table 1. A total of 1249 reports were identified for the analysis of *S. pneumoniae* antibiotic resistance in Iran. Finally, 58 articles (50 in English and 8 in Persian) were included in the study (Figure 1). Disk diffusion, E-test, and broth micro- and macrodilution were the most common methods used for antimicrobial susceptibility testing in the included articles.

### 3.2. Total *S. pneumoniae* Drug Resistance in Iran

**Figure 2:**
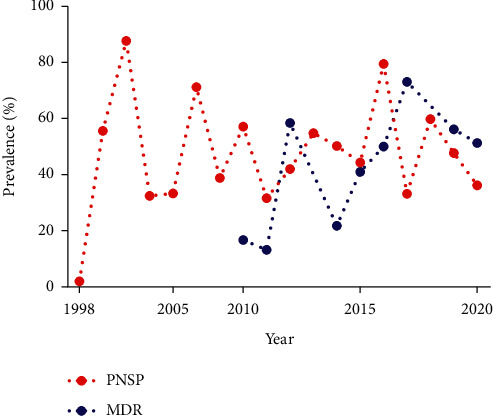
Total trend in the prevalence rate of PNSP and MDR strains in all age groups in Iran over time (based on the year of publication).

The pooled prevalence of *S. pneumoniae* resistance to various antibiotics including erythromycin, azithromycin, tetracycline, levofloxacin, rifampin, clindamycin, vancomycin, trimethoprim/sulfamethoxazole, chloramphenicol, ceftriaxone, amoxicillin, ciprofloxacin, imipenem, linezolid, and cefotaxime was 41.1% (95% CI: 32.9–49.7; *I*^2^ = 93%; *Q* = 545.1; *p*=0.00), 53.2% (95% CI: 38.9–67.1; *I*^2^ = 92.4%; *Q* = 118.4; *p*=0.00), 39.9% (95% CI: 30.2–50.4; *I*^2^ = 95%; *Q* = 506.8; *p*=0.00), 1.7% (95% CI: 0.2–11.1; *I*^2^ = 90.9%; *Q* = 110; *p*=0.00), 1.2% (95% CI: 0.1–13.2; *I*^2^ = 91.1%; *Q* = 67.6; *p*=0.00), 31.7% (95% CI: 20.7–45.2; *I*^2^ = 91.9%; *Q* = 172.8; *p*=0.00), 1.7% (95% CI: 0.7–4.1; *I*^2^ = 85.8%; *Q* = 218.4; *p*=0.00), 63.9% (95% *CI*: 52.3–74; *I*^2^ = 94.6%; *Q* = 672.5; *p*=0.00), 20% (95% CI: 14.2–27.3; *I*^2^ = 91.4%; *Q* = 303.5; *p*=0.00), 10.9% (95% CI: 6.6–17.6; *I*^2^ = 84.7%; *Q* = 130.8; *p*=0.00), 30.5% (95% CI: 12.8–56.8; *I*^2^ = 95.2%; *Q* = 187.6; *p*=0.00), 8.3% (95% CI: 3.6–17.7; *I*^2^ = 89.6%; *Q* = 154.3; *p*=0.00), 6.1% (95% CI: 0.1–89.4; *I*^2^ = 91.8%; *Q* = 36.6; *p*=0.00), 0%, and 8.3% (95% CI: 3.7–17.4; *I*^2^ = 92.5%; *Q* = 189; *p*=0.00), respectively. The frequency of MDR *S. pneumoniae* strains in Iran was 45.3% (95% CI: 34.3–56.8; *I*^2^ = 91.3%; *Q* = 150.7; *p*=0.00). As illustrated in Figure 2, the prevalence of MDR *S. pneumoniae* in Iran showed an increasing trend from 16.7% in 2010 to 51.3% in 2020. A random-effects model was used to estimate pooled effect in terms of the heterogeneity among studies.

### 3.3. *S. pneumoniae* Drug Resistance in Different Provinces of Iran

The results of the subgroup analysis of the prevalence of *S. pneumoniae* antibiotic resistance based on the different geographic locations in Iran are shown in Table 2. A random-effects model was used to combine studies within each subgroup and obtain the overall effect. The highest rates of *S. pneumoniae* antibiotic resistance among different provinces in Iran were as follows: 74.4% to erythromycin in Ardabil, 72.1% to azithromycin in Ardabil, 50% to tetracycline in Khuzestan, 24.2% to levofloxacin in Fars, 41% to rifampin in Kermanshah, 50.1% to clindamycin in Tehran, 2.5% to vancomycin in Hamadan, 96.9% to trimethoprim/sulfamethoxazole in Isfahan, 66.7% to chloramphenicol in Qazvin, 60% to ceftriaxone in Isfahan, 60% to amoxicillin in Isfahan, 20.1% to ciprofloxacin in Fars, 99.1% to imipenem in Hamadan, and 60% to cefotaxime in Isfahan. In addition, the highest rates of PNSP and MDR *S. pneumoniae* strains were detected in Ardabil (95.3% and 74.4%, respectively).

### 3.4. *S. pneumoniae* Drug Resistance in Iranian Children

The results of subgroup analysis based on the age group indicated that 27 studies investigated the prevalence of *S. pneumoniae* antibiotic resistance profiles in Iranian children. Based on the current meta-analysis, *S. pneumoniae* resistance to different antibiotics was as follows: 38.5% (95% CI: 25.7–53.2; *I*^2^ = 93.4%; *Q* = 290.2; *p*=0.00) to erythromycin, 66.5% (95% CI: 54.8–76.5; *I*^2^ = 81%; *Q* = 26.4; *p*=0.00) to azithromycin, 33% (95% CI: 20.2–49; *I*^2^ = 95.9%; *Q* = 223.1; *p*=0.00) to tetracycline, 0.8% (95% CI: 0.3–2.1; *I*^2^ = 0.0%; *Q* = 1.9; *p*=0.92) to levofloxacin, 1.2% (95% CI: 0.1–13.2; *I*^2^ = 91.1%; *Q* = 67.6; *p*=0.00) to rifampin, 17.3% (95% CI: 7.3–35.6; *I*^2^ = 86.8%; *Q* = 45.5; *p*=0.00) to clindamycin, 1.7% (95% CI: 0.4–7.1; *I*^2^ = 90.7%; *Q* = 151; *p*=0.00) to vancomycin, 63.7% (95% CI: 48.3–76.7; *I*^2^ = 94.8%; *Q* = 407.2; *p*=0.00) to trimethoprim/sulfamethoxazole, 17.7% (95% CI: 11.4–26.3; *I*^2^ = 86.1%; *Q* = 93.6; *p*=0.00) to chloramphenicol, 12.6% (95% CI: 6.5–22.9; *I*^2^ = 84.9%; *Q* = 73; *p*=0.00) to ceftriaxone, 35.1% (95% CI: 12.3–67.6; *I*^2^ = 96.3%; *Q* = 162.4; *p*=0.00) to amoxicillin, 5.5% (95% CI: 1.1–22.7; *I*^2^ = 90.7%; *Q* = 53.7; *p*=0.00) to ciprofloxacin, 0.7% (95% CI: 0.0–9.7; *I*^2^ = 0.0%; *Q* = 0.0; *p*=1.00) to imipenem, 0% to linezolid, and 8.3% (95% CI: 3.2–19.8; *I*^2^ = 88.6%; *Q* = 61.4; *p*=0.00) to cefotaxime. Besides, 57.4% (95% CI: 33.1–78.6; *I*^2^ = 91%; *Q* = 44.8; *p*=0.00) of *S. pneumoniae* isolated from Iranian children were MDR strains. Random- or fixed-effects models were used to estimate pooled effect.

### 3.5. Penicillin-Nonsusceptible *S. pneumoniae* in Iran

**Figure 3:**
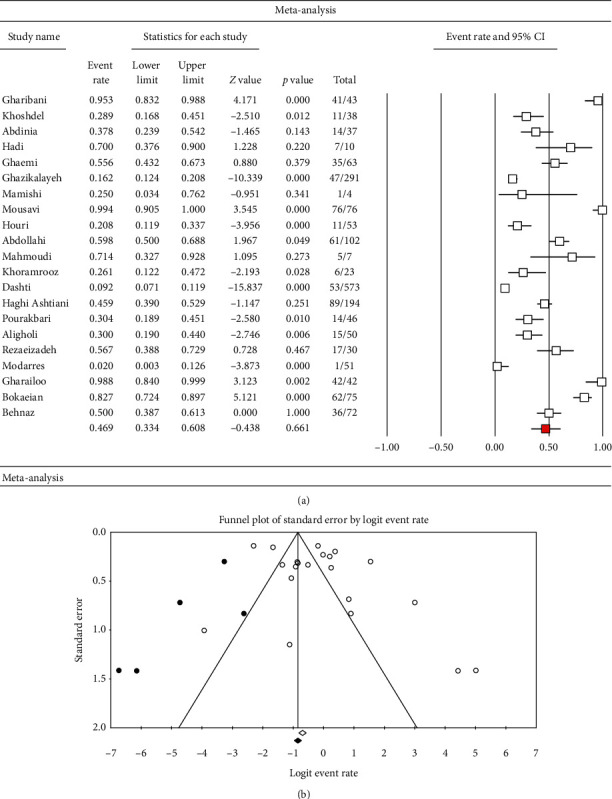
Forest plot (a) and funnel plot (b) showing the prevalence of penicillin-nonsusceptible *S. pneumoniae* in Iranian children.

According to the random-effects model (*I*^2^ = 93.6%; *Q* = 712.6; *p*=0.00), the total prevalence of PNSP strains in Iran was 46.9% (95% CI: 38.6–55.4). In addition, the rate of PNSP strains isolated from Iranian children was 46.9% (95% CI: 33.4–60.8; *I*^2^ = 94.4%; *Q* = 363.1; *p*=0.00) as well (Figure 3(a)). As shown in Figure 3(b), publication bias was detected in the current study due to the evidence of asymmetry in the funnel plot whereas the results of Begg's (*z* = 0.21, *p*=0.83) and Egger's tests (*t* = 1.86, *p*=0.07) were not statistically significant. Finally, as presented in Figure 2, we assessed the frequency of PNSP strains from 1998 to 2020. Figure 2 shows an increasing trend of PNSP strains in Iran.

## 4. Discussion

Antibiotic resistance is consistently growing and has become a global public health crisis. According to the European Center for Disease Prevention and Control (ECDC) and the Center for Disease Control and Prevention (CDC), antibiotic-resistant bacteria in Europe and the USA are associated with an annual mortality rate of 25,000 and 23,000, respectively. Also, nearly 700,000 deaths worldwide are due to antibiotic resistance [5, 67]. It is estimated that antimicrobial resistance will lead to 10 million deaths a year by 2050 [5, 67]. Factors such as antibiotic overuse/misuse in humans and also in the food/veterinary industry along with reduced development of new antibiotics play key roles in the incidence of both Gram-positive and Gram-negative resistant bacteria [5]. Penicillin-resistant *S. pneumonia* was first detected in Australia in 1967. PNSP strains are listed as one of the most serious emerging bacterial threats as of 2017 [3, 6]. The current rate of penicillin-resistant *S. pneumoniae* in Iran is 46.9%, whereas it is found to be 1–5% in the UK, Germany, Austria, Norway, and Sweden, 5–10% in Italy, 10–25% in Portugal, Ireland, Finland, and Turkey, 25–50% in Spain, France, Greece, and Israel, 20% in Brazil, and 66.4% in China [68–70]. The results of subgroup analysis based on the age group showed a similarly high rate of PNSP among Iranian children (46.9%) which could be due to the common use of antibiotics in these patients [39]. Therefore, the prescription of penicillin as the first-choice antibiotic in the treatment of *S. pneumoniae* infections such as meningitis and pneumonia should be avoided. The prevalence of PNSP isolates in Iran has shown a rising trend from 1998 to 2020 (Figure 2). While there was high pneumococcal resistance to amoxicillin in Iran, resistance to other beta-lactam antibiotics such as cephalosporins and carbapenems was rather low. Thus, the extended-spectrum cephalosporins are suitable alternative drugs in the treatment of penicillin-resistant infections including pneumococcal meningitis in Iran. Modification of penicillin-binding proteins (PBPs) particularly PBP1a, PBP2x, and PBP2b as well as mutations in *cpoA*, *ciaH*, *murM*, and *murN* genes has been described as the main mechanisms of resistance in *S. pneumoniae* to beta-lactam antibiotics [3]. Pneumococcal resistance to macrolides, fluoroquinolones, and tetracyclines has also been reported [1]. The prevalence of macrolide-resistant *S. pneumoniae* is geographically variable as it ranges from 25 to 50% in France, Italy, and Greece, 10 to 25% in Spain, Portugal, the UK, Germany, Poland, Norway, and Finland, and 1 to 5% in Latvia and Sweden [68]. In Iran, 41.1% and 53.2% of *S. pneumoniae* isolates were resistant to erythromycin and azithromycin, respectively. Ribosomal modification, efflux system, and point mutations are involved in the emergence of macrolide-resistant *S. pneumoniae* [3]. An important mechanism of *S. pneumoniae* resistance to clindamycin is the alteration of the ribosomal target through *erm(B)* gene which encodes a 23S RNA methylase [71]. Clindamycin has shown a strong activity against community-acquired infections of *S. pneumoniae* [71]. However, the rates of clindamycin-resistant pneumococcal strains in the current study were high (31.7%) and included 25% in Egypt, 35.1% in Turkey, and 21.8% in the United States [3,71]. Penicillins and macrolides have been largely applied in the treatment of community-acquired pneumonia and other respiratory tract infections by *S. pneumoniae* [72]. However, a high resistance rate to these antibiotics has led to the use of quinolones against important bacterial respiratory tract pathogens [72]. Hence, a combination of vancomycin and gentamicin is proposed for treating infections caused by penicillin- and cephalosporin-resistant *S. pneumoniae* strains [3]. The findings of the present study on the prevalence of fluoroquinolone-resistant strains of *S. pneumoniae* indicated a low resistance rate to levofloxacin (1.7%) and ciprofloxacin (8.3%) in Iran. The prevalence of fluoroquinolone-resistant pneumococcal strains in other countries was as follows: 4% in Egypt, 1.8% in Turkey, 1-2% in the USA, and <10% in Belgium [3, 71, 72]. A low incidence of vancomycin-resistant *S. pneumoniae* was found in Iran (1.7%), and no resistance has been reported in many other countries [3]. Factors associated with resistance to fluoroquinolones in clinical pneumococcal isolates include mutations in the quinolone-resistance-determining regions (QRDRs) of *gyrA*, *gyrB*, *parC*, and *parE* genes as well as the overexpression of *pmrA* gene (codes for an efflux pump) and *patA* and *patB* genes (code for an ABC transporter) [3, 71]. Point mutation in a histidine kinase gene (*vncS*) is associated with the emergence of vancomycin-tolerant pneumococcal strains [3]. The highest drug resistance rate among pneumococcal isolates in Iran was observed to folate pathway inhibitors (i.e., trimethoprim/sulfamethoxazole (63.9%)). Cotrimoxazole-resistant *S. pneumoniae* were isolated in 25–45% of strains in the USA, 55% in Egypt, 100% in Saudi Arabia, and 67.2% in Turkey [3, 71]. Mutations in dihydrofolate reductase (DHFR) and in dihydropteroate synthase (DHPS) are the mechanisms of resistance to folate inhibitors [3, 71]. Studies from the Middle East and the USA have reported a high rate of *S. pneumoniae* resistance to tetracycline which could be attributed to extensive use of this antimicrobial agent [3, 71]. A similar result was observed in the current study (39.9%). Resistance to chloramphenicol (a bacterial protein inhibitor) was high whereas there was no resistance to linezolid. Two other important findings of the study included a high prevalence of MDR pneumococci in Iranian people (45.3%), especially children (57.4%) with a rising trend from 2010 to 2020 (Figure 2), and also the isolation of *S. pneumoniae* resistant to many drugs (such as erythromycin, azithromycin, tetracycline, trimethoprim/sulfamethoxazole, and amoxicillin) in Iranian children. Available data from CDC showed that MDR *S. pneumoniae* is responsible for more than 30% of invasive pneumococcal disease throughout the United States [73, 74]. Therefore, timely vaccination in Iranian children and ongoing surveillance on drug resistance trend along with the use of combination therapy or the use of newer antibiotics are needed to improve microorganism susceptibility.

## 5. Conclusion

The current study indicated a high prevalence of PNSP and MDR strains in Iran among all age groups. Similar results were also observed in the frequency of erythromycin-, azithromycin-, tetracycline-, clindamycin-, trimethoprim/sulfamethoxazole-, chloramphenicol-, and amoxicillin-resistant *S. pneumoniae* strains. These findings could be due to the high consumption of nonprescribed antibiotics in Iran. Hence, strategies to prevent emerging drug-resistant pneumococcal infections and treatment failure in Iran include (1) continuous regional monitoring of nasopharyngeal carriers of antibiotic-resistant *S. pneumoniae* in children, (2) controlled administration of antibiotics to improve microorganism susceptibility, (3) use of combination therapies or drugs with low resistance rate in accordance with local resistance patterns, and (4) identification of the most common pneumococcal serotypes and their drug resistance rates in Iranian population to produce effective pneumococcal vaccines. The most effective antibiotics for the treatment of pneumococcal infections in Iran based on the current study are levofloxacin, rifampin, vancomycin, ceftriaxone, ciprofloxacin, imipenem, linezolid, and cefotaxime.

## Figures and Tables

**Table 1:** Extracted information from eligible studies included in the meta-analysis.

Author (ref)	Province	Published time	Enrollment time	Age group	Strain (*n*)	AST	Antibiotic resistance (*n*)																
							PNSP	ERY	AZM	TET	LVX	RIF	CLI	VAN	SXT	CHL	CRO	AMX	CIP	IPM	LZD	CTX	MDR
Gharibani et al. [8]	Ardabil	2019	2015	Children	43	Disk diffusion	41	32	31	18	0	0	12	0	35	7	ND	ND	ND	ND	ND	ND	32
Khoshdel et al. [9]	Chaharmahal and Bakhtiari	2009	2007	Children	38	Disk diffusion	11	ND	ND	ND	ND	ND	ND	ND	ND	ND	ND	ND	ND	ND	ND	ND	ND
Imani et al. [10]	Chaharmahal and Bakhtiari	2007	2005	ND	17	Broth microdilution	16	10	ND	ND	ND	ND	ND	ND	ND	ND	1	ND	2	ND	ND	ND	ND
Abdinia et al. [11]	East Azerbaijan	2014	2003–2013	Children	37	Disk diffusion	14	6	ND	ND	ND	1	ND	0	12	2	4	ND	3	ND	ND	3	ND
Hadi and Bagheri [12]	Fars	2019	2011–2016	Children	10	Disk diffusion	7	3	ND	ND	ND	ND	3	0	9	1	4	ND	ND	ND	ND	ND	ND
Kargar et al. [13]	Fars	2015	2011–2012	Children/adult	45	Disk diffusion	ND	ND	ND	ND	31	ND	ND	ND	ND	ND	ND	ND	40	ND	ND	ND	ND
Kargar et al. [14]	Fars	2012	2010–2011	Children/adult	50	Disk diffusion	30	28	22	5	2	ND	ND	ND	24	0	ND	ND	ND	ND	ND	25	30
Shishegar et al. [15]	Fars	2011	2007–2008	Children	10	Disk diffusion	ND	1	ND	ND	ND	ND	ND	ND	10	ND	2	4	0	ND	ND	2	ND
Japoni et al. [16]	Fars	2010	2005–2006	ND	13	Disk diffusion	5	3	ND	ND	ND	ND	1	0	6	1	ND	ND	1	ND	ND	ND	ND
Kohanteb and Sadeghi [17]	Fars	2007	ND	Children/adult	115	Broth microdilution	39	21	15	28	ND	ND	ND	0	ND	9	7	ND	9	ND	ND	5	ND
Ghaemi et al. [18]	Golestan	2002	1998–1999	Children	63	Disk diffusion	35	5	ND	23	ND	ND	13	ND	ND	ND	ND	ND	ND	ND	ND	ND	ND
Khademi et al. [19]	Hamadan	2016	2013–2014	Children/adult	6	Disk diffusion	ND	ND	ND	ND	ND	ND	ND	0	3	ND	0	ND	1	ND	ND	0	ND
Yousefi Mashouf et al. [20]	Hamadan	2014	2009–2013	Children	35	Disk diffusion	ND	ND	ND	ND	ND	ND	ND	ND	9	12	ND	19	ND	ND	ND	ND	ND
Yousefi Mashouf et al. [21]	Hamadan	2003	1998–2000	Children	11	Disk diffusion	ND	ND	ND	ND	ND	ND	ND	ND	3	4	ND	6	ND	ND	ND	ND	ND
Mosleh et al. [22, 23]	Hamadan	2014	ND	ND	55	E-test	52	14	10	ND	ND	ND	ND	0	ND	ND	ND	ND	6	55	ND	ND	12
Yeganeh-Moghadam et al. [24]	Isfahan	2014	2011–2013	Children	15	Disk diffusion	ND	5	9	ND	ND	ND	ND	ND	15	ND	9	9	0	ND	ND	9	ND
Ghazikalayeh et al. [25]	Isfahan	2014	2011–2012	Children	291	Disk diffusion	47	10	ND	74	0	0	5	0	ND	ND	ND	ND	ND	ND	0	ND	ND
Sabory et al. [26]	Kermanshah	2015	2012	Children	83	Disk diffusion	ND	ND	53	ND	ND	34	ND	ND	31	ND	3	47	ND	ND	ND	ND	34
Khosravi et al. [27]	Khuzestan	2007	2005–2006	Adult	6	Disk diffusion	ND	ND	ND	3	ND	ND	ND	ND	0	ND	3	ND	ND	ND	ND	ND	ND
Moafi and Issazadeh [28]	Qazvin	2016	2013–2014	Children	6	Disk diffusion	ND	ND	ND	ND	ND	ND	ND	ND	4	4	ND	ND	ND	ND	ND	ND	ND
Mamishi et al. [29]	Tehran	2019	2017–2018	Children	4	Disk diffusion	1	4	ND	ND	ND	ND	2	0	1	ND	ND	ND	ND	ND	ND	ND	ND
Ghahfarokhi et al. [30]	Tehran	2020	2015–2019	Children/adult	80	Disk diffusion	29	49	ND	31	ND	ND	47	0	57	15	13	ND	ND	ND	ND	ND	41
Azarsa et al. [31]	Tehran	2019	2015	Children/adult	46	Disk diffusion	12	ND	ND	ND	ND	ND	ND	ND	ND	ND	ND	ND	ND	ND	ND	ND	ND
Mousavi et al. [32]	Tehran	2017	2014–2015	Children	76	Disk diffusion	76	65	65	4	ND	0	ND	ND	ND	ND	ND	ND	ND	ND	ND	ND	76
Ahmadi et al. [33]	Tehran	2019	2013–2016	Children/adult	100	Disk diffusion	22	59	ND	57	ND	ND	49	ND	92	23	ND	ND	3	ND	ND	ND	54
Houri et al. [34]	Tehran	2017	2013–2016	Children	53	Broth microdilution	11	15	ND	13	0	ND	ND	0	12	ND	4	ND	ND	ND	0	3	9
Talebi et al. [35]	Tehran	2019	2013–2015	ND	161	Disk diffusion	93	96	ND	121	ND	ND	84	ND	151	95	ND	ND	ND	ND	ND	ND	69
Moghadam et al. [36]	Tehran	2017	2013–2015	Children/adult	100	Disk diffusion	26	64	ND	77	0	ND	56	0	96	44	0	ND	ND	0	0	ND	71
Farshad et al. [37]	Tehran	2016	2013	Adult	48	Disk diffusion	40	ND	ND	ND	ND	ND	ND	ND	ND	ND	ND	ND	ND	ND	ND	ND	ND
Rahbar et al. [38]	Tehran	2019	2012–2016	Children/adult	50	E-test	15	36	35	28	1	ND	26	0	32	13	ND	ND	ND	ND	ND	ND	ND
Tabatabaei et al. [39]	Tehran	2017	2012–2015	Children/adult	73	Broth microdilution	16	61	ND	ND	ND	ND	ND	0	11	ND	23	ND	ND	ND	ND	31	ND
Talebi et al. [40]	Tehran	2016	2011–2013	Children/adult	100	Disk diffusion	28	60	ND	85	ND	ND	78	0	93	48	0	0	ND	0	0	0	50
Azadegan et al. [41]	Tehran	2015	2011–2013	Children/adult	186	Disk diffusion	ND	88	ND	186	ND	ND	ND	ND	ND	ND	ND	ND	ND	ND	ND	ND	ND
Abdollahi et al. [42]	Tehran	2018	2011–2012	Children	102	Disk diffusion	61	24	ND	11	0	ND	ND	0	70	23	ND	ND	ND	ND	ND	0	ND
Ahmadi et al. [43]	Tehran	2013	2011	Children/adult	88	Disk diffusion	ND	47	ND	55	ND	ND	ND	ND	ND	ND	ND	ND	ND	ND	ND	ND	ND
Soltan Dallal et al. [44]	Tehran	2013	2011	Children/adult	15	Disk diffusion	12	10	ND	0	ND	ND	ND	8	ND	8	ND	12	ND	ND	ND	ND	ND
Sadeghi et al. [45]	Tehran	2015	2010–2012	Children/adult	80	E-test	36	ND	ND	ND	ND	ND	ND	ND	ND	ND	ND	ND	ND	ND	ND	ND	ND
Ahmadi et al. [46]	Tehran	2015	2010–2012	Children/adult	70	Disk diffusion	60	ND	ND	ND	ND	ND	ND	ND	ND	ND	ND	ND	ND	ND	ND	ND	ND
Mahmoudi et al. [47]	Tehran	2013	2009–2011	Children	7	Disk diffusion	5	ND	ND	ND	ND	ND	ND	ND	7	ND	4	ND	ND	ND	ND	ND	ND
Khoramrooz et al. [48]	Tehran	2012	2009–2010	Children	23	Disk diffusion	6	11	13	ND	0	ND	8	0	23	ND	3	ND	0	ND	0	ND	ND
Rahimi et al. [49]	Tehran	2015	2008–2012	Children/adult	38	NA	3	2	ND	8	ND	ND	ND	2	29	4	ND	ND	6	ND	ND	ND	ND
Habibian et al. [50]	Tehran	2013	2008–2012	ND	50	Broth microdilution	9	ND	ND	ND	ND	ND	ND	4	ND	ND	ND	ND	2	ND	ND	ND	ND
Dashti et al. [51]	Tehran	2012	2008–2009	Children	573	Disk diffusion	53	ND	315	377	ND	0	ND	9	68	34	26	23	9	ND	ND	17	ND
Ashtiani et al. [52]	Tehran	2014	2001–2011	Children	194	Disk diffusion	89	99	ND	ND	ND	ND	ND	0	139	58	23	ND	ND	ND	ND	25	ND
Pourakbari et al. [53]	Tehran	2012	2001–2005	Children	46	Disk diffusion	14	29	ND	ND	ND	ND	3	ND	38	8	ND	ND	ND	ND	ND	ND	ND
Aligholi et al. [54]	Tehran	2009	2001–2005	Children	50	Agar dilution	15	29	ND	ND	ND	0	ND	0	ND	ND	0	ND	15	ND	ND	ND	ND
Oskoui et al. [55]	Tehran	2010	2000–2008	ND	54	Disk diffusion	38	9	ND	10	ND	ND	ND	ND	28	ND	ND	ND	ND	ND	ND	2	9
Jahanmehr et al. [56]	Tehran	2004	1999–2001	ND	66	Disk diffusion	12	ND	ND	ND	ND	ND	ND	0	ND	ND	ND	12	ND	ND	ND	ND	ND
Oskoui et al. [57]	Tehran	2003	1998–2000	ND	130	Disk diffusion	114	10	ND	47	ND	ND	ND	0	57	28	ND	ND	ND	ND	ND	ND	ND
Rezaeizadeh et al. [58]	Tehran	2012	1998–2008	Children	30	Disk diffusion	17	6	ND	ND	ND	ND	ND	0	19	6	ND	ND	ND	ND	ND	ND	ND
Modarres et al. [59]	Tehran	1998	1993–1995	Children	51	Disk diffusion	1	1	ND	ND	ND	ND	ND	29	34	2	ND	ND	ND	ND	ND	ND	ND
Gharailoo et al. [60]	Sistan and Balouchastan	2016	2013–2014	Children	42	Disk diffusion	42	23	ND	26	0	ND	ND	0	39	6	ND	7	ND	ND	ND	0	ND
Bokaeian et al. [61]	Sistan and Balouchastan	2012	2008–2010	Children	75	Disk diffusion	62	66	ND	43	0	ND	ND	0	47	12	0	ND	ND	0	ND	ND	43
Bokaeian et al. [62]	Sistan and Balouchastan	2011	2007–2008	Children/adult	136	Broth microdilution	43	25	ND	13	ND	ND	ND	0	ND	11	5	ND	2	ND	ND	3	18
Rahbar et al. [63]	West Azerbaijan	2005	1999–2001	ND	24	Disk diffusion	8	ND	ND	ND	ND	ND	0	0	0	ND	ND	ND	0	ND	ND	ND	ND
Behnaz et al. [64]	Yazd	2004	2002	Children	72	Disk diffusion	36	45	ND	22	ND	ND	ND	ND	45	ND	ND	ND	ND	ND	ND	ND	ND
Karami et al. [65]	Zanjan	2009	2006–2007	Children/adult	57	Broth macrodilution	33	ND	ND	ND	ND	ND	ND	0	ND	ND	ND	ND	ND	ND	ND	ND	ND

PNSP: penicillin-nonsusceptible *S. pneumoniae* (intermediately resistant and fully resistant); ERY: erythromycin; AZM: azithromycin; TET: tetracycline; LVX: levofloxacin; RIF: rifampin; CLI: clindamycin; VAN: vancomycin; SXT: trimethoprim/sulfamethoxazole; CHL: chloramphenicol; CRO: ceftriaxone; AMX: amoxicillin; CIP: ciprofloxacin; IPM: imipenem; LZD: linezolid; CTX: cefotaxime; MDR: multidrug-resistant (resistant to ≥3 antibiotic classes); AST: antimicrobial susceptibility testing; ND: not determined.

**Table 2:** *S. pneumoniae* antibiotic resistance profiles in different provinces of Iran.

Province	Antibiotic resistance (%)																
	PNSP	ERY	AZM	TET	LVX	RIF	CLI	VAN	SXT	CHL	CRO	AMX	CIP	IPM	LZD	CTX	MDR
Ardabil	95.3	74.4	72.1	41.9	1.1	1.1	27.9	1.1	81.4	16.3	ND	ND	ND	ND	ND	ND	74.4
Chaharmahal and Bakhtiari	69.2	58.8	ND	ND	ND	ND	ND	ND	ND	ND	5.9	ND	11.8	ND	ND	ND	ND
East Azerbaijan	37.8	16.2	ND	ND	ND	2.7	ND	1.3	32.4	5.4	10.8	ND	8.1	ND	ND	8.1	ND
Fars	48.6	27.4	25.5	17	24.2	ND	18.5	1.9	66.2	7.3	17.1	40	20.1	ND	ND	18.6	60
Golestan	55.6	7.9	ND	36.5	ND	ND	20.6	ND	ND	ND	ND	ND	ND	ND	ND	ND	ND
Hamadan	94.5	25.5	18.2	ND	ND	ND	ND	2.5	29.1	34.8	7.1	54.3	11.6	99.1	ND	7.1	21.8
Isfahan	16.2	11.4	60	25.4	0.2	0.2	1.7	0.2	96.9	ND	60	60	3.1	ND	0	60	ND
Kermanshah	ND	ND	63.9	ND	ND	41	ND	ND	37.3	ND	3.6	56.6	ND	ND	ND	ND	41
Khuzestan	ND	ND	ND	50	ND	ND	ND	ND	7.1	ND	50	ND	ND	ND	ND	ND	ND
Qazvin	ND	ND	ND	ND	ND	ND	ND	ND	66.7	66.7	ND	ND	ND	ND	ND	ND	ND
Tehran	40.2	47.3	68.1	45.7	1.1	0.4	50.1	2.2	69.5	24.1	9.8	12.8	5.9	0.5	0	5.4	47
Sistan and Balouchastan	79.3	55.4	ND	38.1	0.9	ND	ND	0.7	81.3	12.1	2.6	16.7	1.5	0.7	ND	2	31.2
West Azerbaijan	33.3	ND	ND	ND	ND	ND	2	0.2	2	ND	ND	ND	2	ND	ND	ND	ND
Yazd	50	62.5	ND	30.6	ND	ND	ND	ND	62.5	ND	ND	ND	ND	ND	ND	ND	ND
Zanjan	57.8	ND	ND	ND	ND	ND	ND	0.9	ND	ND	ND	ND	ND	ND	ND	ND	ND

## Data Availability

There are no raw data associated with this systematic review and meta-analysis.
